# The glycolytic pathway of *Trimastix pyriformis *is an evolutionary mosaic

**DOI:** 10.1186/1471-2148-6-101

**Published:** 2006-11-23

**Authors:** Alexandra Stechmann, Manuela Baumgartner, Jeffrey D Silberman, Andrew J Roger

**Affiliations:** 1 Department of Biochemistry and Molecular Biology, Dalhousie University, Sir Charles Tupper Building, Halifax, Canada; 2 Department für Biologie I, Botanik, Ludwig-Maximilians-Universität München, Menzingerstraße 67, D-80638 München, Germany; 3 Department of Biological Sciences, University of Arkansas, Fayetteville, AR 72701, USA; 4 Canadian Institute for Advanced Research, Evolutionary Biology Program, Dalhousie University, Sir Charles Tupper Building, Halifax, Canada

## Abstract

**Background:**

Glycolysis and subsequent fermentation is the main energy source for many anaerobic organisms. The glycolytic pathway consists of ten enzymatic steps which appear to be universal amongst eukaryotes. However, it has been shown that the origins of these enzymes in specific eukaryote lineages can differ, and sometimes involve lateral gene transfer events. We have conducted an expressed sequence tag (EST) survey of the anaerobic flagellate *Trimastix pyriformis *to investigate the nature of the evolutionary origins of the glycolytic enzymes in this relatively unstudied organism.

**Results:**

We have found genes in the *Trimastix *EST data that encode enzymes potentially catalyzing nine of the ten steps of the glycolytic conversion of glucose to pyruvate. Furthermore, we have found two different enzymes that in principle could catalyze the conversion of phosphoenol pyruvate (PEP) to pyruvate (or the reverse reaction) as part of the last step in glycolysis. Our phylogenetic analyses of all of these enzymes revealed at least four cases where the relationship of the *Trimastix *genes to homologs from other species is at odds with accepted organismal relationships. Although lateral gene transfer events likely account for these anomalies, with the data at hand we were not able to establish with confidence the bacterial donor lineage that gave rise to the respective *Trimastix *enzymes.

**Conclusion:**

A number of the glycolytic enzymes of *Trimastix *have been transferred laterally from bacteria instead of being inherited from the last common eukaryotic ancestor. Thus, despite widespread conservation of the glycolytic biochemical pathway across eukaryote diversity, in a number of protist lineages the enzymatic components of the pathway have been replaced by lateral gene transfer from disparate evolutionary sources. It remains unclear if these replacements result from selectively advantageous properties of the introduced enzymes or if they are neutral outcomes of a gene transfer 'ratchet' from food or endosymbiotic organisms or a combination of both processes.

## Background

Eukaryotes catabolize glucose to pyruvate via the Embden-Meyerhof-Parnas (EMP) glycolytic pathway [1]. A number of protists are known that lack classical mitochondrial structures and electron-transport-linked ATP production, instead relying on substrate-level phosphorylation. In these organisms, glycolysis and subsequent fermentation reactions play a central role in their energy metabolism [2-4]. Although often referred to as 'amitochondriates', most of these protist taxa have been shown to harbor organelles of mitochondrial descent [5-7]. Hydrogenosomes for example metabolize pyruvate via a fermentative pathway that yields ATP and hydrogen [3]. Other mitochondrion-derived organelles, such as the 'mitosomes' of the parasites *Entamoeba histolytica *and *Giardia lamblia*, seem to have lost all core metabolic functions [7]. In these organisms, pyruvate is catabolized by fermentation in the cytosol.

Although the core EMP pathway appears to be almost universal amongst eukaryotes, amitochondriate protists have been shown to be surprisingly flexible in terms of the specific enzyme families they utilize to carry out glycolytic reactions. More interestingly these enzymes appear to have rather diverse origins [1,8] and it is likely that this variability was achieved, at least in part, by lateral gene transfer (LGT) [9-15]. For instance, in the first step of glycolysis, diplomonads and parabasalids use glucokinase, rather than hexokinase which carries out this reaction in most other eukaryotes [11,13]. Similarly, amitochondriate protists such as parabasalids, diplomonads, pelobionts and entamoebids cleave fructose-1,6-bisphosphate with a class II fructose-bisphosphate aldolase (FBA), while most other eukaryotes possess a class I FBA [14,16-18]. These two enzymes are non-homologous and belong to different superfamilies [1,16]. In many cases, the glycolytic enzymes that are replaced by LGT appear to come from a bacterial donor lineage. For instance, diplomonad and parabasalid glucose phosphate isomerases (GPI) are more closely related to the GPIs of cyanobacteria and chloroplasts than to cytosolic GPIs of other eukaryotes [11]. The glyceraldehyde-3-phosphate dehydrogenase (GAPDH) of parabasalids is not part of the GapC clade which encompasses most eukaryotic GAPDHs including those of most amitochondriates. Instead, it clusters most closely with the sequences of the spirochaete genus *Borrelia *within the bacterial GapAB clade [10,19,20]. Similarly, the evolution of phosphofructokinase (PFK) shows phospho-donor changes and frequent lateral gene transfer [15]. In amitochondriates, pyrophosphate-dependent, ATP-dependent or both types of PFK are present – most of them acquired by LGT [9,12,15].

In some protist taxa, additional enzymes involved in glycolytic reactions are found that are rare amongst most eukaryotes. For instance, pyruvate phosphate dikinase (PPDK) is an analogous enzyme to pyruvate kinase (PK), with a key difference being that PPDK uses pyrophosphate instead of ATP to catalyze the conversion of phosphoenolpyruvate into pyruvate and generates ATP in the process. PPDK is present in a number of eukaryotes including the anaerobes *Entamoeba histolytica*, *Giardia lamblia *[21,22], *Trichomonas vaginalis *and *Streblomastix strix *[23] and the phylogenetic affinites of the eukaryote enzymes are diverse [23].

These examples demonstrate the diversity in the equipment of glycolytic enzymes and in their evolutionary history in protists in general, and anaerobic species in particular. However, our knowledge of the biochemistry of diverse protist lineages still remains severely limited to taxa of biomedical or agricultural importance.

Here we expand this knowledge to include the anaerobic flagellate *Trimastix pyriformis *[24]. Species of the genus *Trimastix *are free-living nanoflagellates which thrive in anoxic environments, where they feed on bacteria [25]. They harbor small double membrane-bound organelles of unknown function which structurally resemble hydrogenosomes [25,26] but are probably derived from mitochondria [27]. Phylogenetic analyses indicate that *Trimastix *species form a sister group to the oxymonads, another goup of 'amitochondriate' protist that are symbionts of termites and cockroaches [28]. The placement of these organisms in the eukaryotic tree remains contentious, but on the basis of ultrastructural comparisons they have been suggested to be part of a eukaryote super-group called the Excavata [29]. The Excavata is comprised of a number of aerobic protist lineages including the Heterolobosea, Euglenozoa (e.g. kinetoplastids) and jakobid flagellates as well as anaerobic lineages such as diplomonads (*Giardia*), *Carpediemonas *and parabasalids (e.g. *Trichomonas*). Although, ultrastructural evidence for the Excavata is convincing [29], molecular phylogenetic support remains weak [30,31].

To improve our knowledge of the biochemical diversity of anaerobic protists we initiated an EST project of *Trimastix pyriformis*. We identified a large number of ESTs coding for enzymes involved in glycolysis and subsequent fermentation. Our phylogenetic analyses reveal that, while some glycolytic enzymes in this organism were inherited by vertical descent, about half of the glycolytic enzymes of *Trimastix *were acquired by lateral gene transfer events, likely from different bacterial donors. Although the glycolytic pathway is universally conserved in eukaryotes, its enzyme components are evolutionarily labile and have been repeatedly replaced in separate eukaryotic lineages by gene transfer from diverse eubacterial donors.

## Results

### Phylogenies of glycolytic enzymes of *Trimastix pyriformis*

From our EST data we identified 9 homologs of glycolytic enzymes typically found in other eukaryotes plus a second enzyme for the final glycolytic step (pyruvate phosphate dikinase (PPDK)). In order to completely sequence each homolog, 5'-RACE and primer walking sequencing were performed. Putative enzyme activities, lengths, and GC contents of the encoded proteins are shown in Table 1. In the following sections we describe the properties and phylogenetic affinities of each enzyme in turn.

**Table 1:** ORFs coding for glycolytic enzymes of *Trimastix pyriformis*: Number of nucleotides, G+C content, and accession number

Enzyme	length of ORF in nucleotides	G+C content in %	accession number
hexokinase	1368	60.16	DQ845789
glucose phosphate isomerase	1647	61.75	DQ845790
fructose bisphosphate aldolase	972	57.61	DQ845791
triose phosphate isomerase	789	64.90	DQ845792
glyceraldehyde phosphate dehydrogenase	1056	61.46	DQ845793
phosphoglycerate kinase	1206	61.28	DQ845794
phosphoglycerate mutase	1701	61.55	DQ845795
enolase	1323	61.53	DQ845796
pyruvate kinase	1482	64.98	DQ845797
pyruvate dikinase	2637	61.32	DQ845798

#### Hexokinase

The evolution of hexokinases is characterized by several independent gene duplications which gave rise to numerous isoenzymes within different phyla: plants have two isoenzymes (hxk1, hxk2), vertebrates have four (hexokinase A-D) and yeasts have two (P_I _and P_II_) [32]; the vertebrate hexokinase D is often referred to as glucokinase. Like most aerobic eukaryotes and the amitochondriate parasite *Entamoeba histolytica*, *Trimastix *appears to use hexokinase for the first enzymatic step in glycolysis whereas the amitochondriate 'excavates' *Giardia intestinalis*, *Spironucleus barkhanus *and *Trichomonas vaginalis *all appear to use glucokinase [11,13]. It is uncertain if bacteria use a real hexokinase, however search of several databases revealed three prokaryotes (*Bacteroides *and 2 spirochaetes) with hexokinases that were readily aligned with the eukaryotic hexokinase genes. These three prokaryotic sequences form a distinct clade that is clearly separated from the eukaryotic hexokinase sequences (see Additional file 1). The eukaryotic part of the tree shows very little resolution between major groups with Fungi appearing to be paraphyletic with respect to bacteria and other eukaryotes, a feature that is not supported by bootstrap analysis. The *Trimastix *homolog emerges firmly within the eukaryotes, but is not strongly allied with any other lineages. Thus the *Trimastix *hexokinase gene is most probably of eukaryotic origin via vertical descent.

#### Glucose Phosphate Isomerase (GPI)

The GPI gene family can be divided into three major types of GPIs which show only a low degree of sequence similarity [11,33]: type I GPIs are found in the eukaryotic cytosol and in many bacteria. Type II are typical for cyanobacteria and chloroplasts but were recently also detected in the amitochondriate excavates *Giardia intestinalis*, *Spironucleus barkhanus*, and *Trichomonas vaginalis *[11]. Finally, the GPIs of the archaeon *Methanococcus jannaschii *and some Gram positive bacteria are assigned to type III [33]. For tree reconstruction, we used only GPIs of type I and II (see Additional file 2). The GPI of *Trimastix pyriformis *is of type I and not similar to the type II GPIs of diplomonads and parabasalids. In the type I subtree, several bacterial lineages and two clades of eukaryotic GPIs can be distinguished (Additional file 2): one of the clades consists of cytosolic GPIs from alveolates, stramenopiles, and green plants. The second comprises cytosolic GPIs of fungi and animals, glycosomal GPIs of kinetoplastids, and a clade of bacterial GPIs (Proteobacteria and Cytophaga). Likelihood comparisons of different possible trees neither supported nor rejected this seemingly robust grouping of animals and bacteria [33] and it was concluded that the branching pattern within the type I subtree is very unstable and currently unresolved. Except for the most basal branches, this also describes our results. The *Trimastix *GPI falls in the unresolved part of the type I subtree (Additional file 2) where it groups with the alpha-proteobacterium *Agrobacterium tumefaciens *(ML analysis only) but with no statistical support. Given the poor resolution of the type I GPI subtree, we cannot trace the origin of the *Trimastix *GPI other than it is quite distinct from the type II forms of the other amitochondriate flagellates (parabasalids and diplomonads).

#### Fructose-1,6-bisphosphate aldolase (FBA)

**Figure 1:**
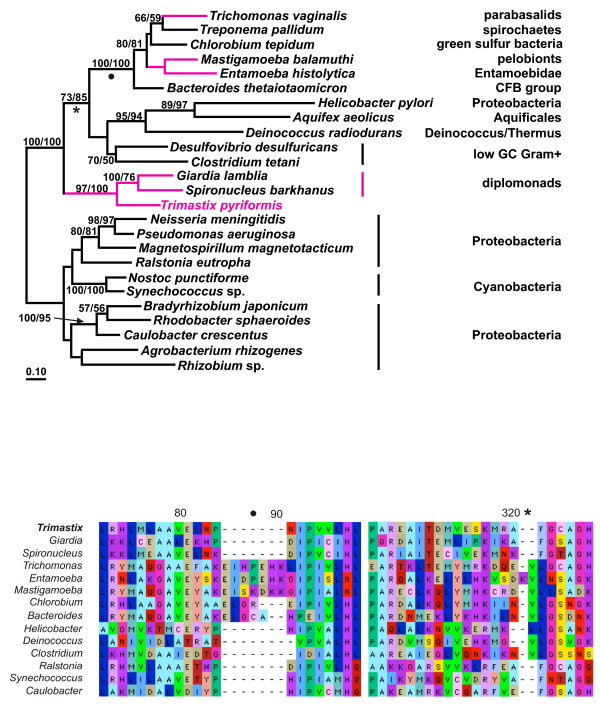
**A – ML tree of FBA protein sequences**. Maximum likelihood (ML) tree of class II type B FBAs based on 268 aligned aa positions. Amitochondriate protists are labelled pink and eubacteria black. The numbers at the bipartitions are ML distance bootstrap values (left: puzzleboot) and ML bootstrap values (right: phyml). Bootstrap values below 50% are omitted. The asterisk and the dot mark indel events in FBA amino acid sequences as shown in the alignment in (1B), supporting indirectly the grouping of *Trimastix *with the diplomonads to the exclusion of the remaining amitochondriates. For display purposes the tree was arbitrarily rooted. **B – FBA amino acid alignment sections showing indel events**. Two separate sections from the FBA alignment which was used to calculate the tree from 1A are shown. The dot marks a 4–7 amino acid insertion: the amitochondriate protists *Trichomonas*, *Mastigamoeba *and *Entamoeba *have a 7 aa insertion; *Treponema *(not shown in the alignment) and *Chlorobium *have a 4 aa insertion and *Bacteroides *has a 5 aa insertion. The insertion event is supported by high bootstrap support in the ML tree (100%). The asterisk marks another indel event, where the three amitochondriate protists *Trimastix*, *Spironucleus *and *Giardia *have a characteristic deletion followed by a phenylalanin seen also in the clade with *Ralstonia*, *Synechococcus *and *Caulobacter*. This deletion separates these two clades from the remaining part of the tree.

A phylogeny of type B class II FBA enzymes is shown in Fig 1A. *Trimastix pyriformis *possesses a type B class II FBA like the other amitochondriates [14,17] and branches robustly with diplomonad FBA sequences in our phylogenetic analysis (Fig. 1A). The other amitochondriates form a clearly separated clade branching within diverse bacteria. An alignment of class II type B FBA protein sequences supports the phylogenetic separation of these groups of amitochondriate taxa; the FBAs of the diplomonads and *Trimastix *share some specific indels to the exclusion of the other amitochondriates (Fig. 1B). However, none of those indels is exclusive to the diplomonad and *Trimastix *sequences but is also found in related FBAs of bacteria (see Fig. 1B and legend). Our tree shows some similarity to an earlier study, where *Mastigamoeba *and *Entamoeba *also group together [14]; in that study *Trichomonas *groups with these two amitochondriates and this monophyly was not rejected by a Shimodaira-Hasegawa test (monophyly of all amitochondriates was rejected) [14]. In our study however, *Trichomonas *groups with *Treponema*, albeit bootstrap support is not very high. Lateral transfer scenarios that account for this particular phylogenetic distribution in eukaryotes are complex, especially when relationships amongst the excavate taxa are concerned. Regardless, our phylogeny and indel data suggest that amitochondrial protists have acquired their FBAs from at least two different sources implying at least two individual LGT events.

#### Triosephosphate isomerase (TPI)

The TPIs of all eukaryotes including amitochondriates are monophyletic (see Additional file 3). In our analysis, the monophyly of major eukaryotic phyla (with representatives from more than one taxon) like animals, fungi, green plants, kinetoplastids, stramenopiles, and diplomonads are recovered although monophyly of fungi is only poorly supported. The relationships between the eukaryotic lineages are not resolved. The amitochondriates *Trimastix pyriformis*, *Trichomonas vaginalis*, the diplomonads, and *Entamoeba histolytica *each branch individually in different parts of the tree. Since there is no clear affiliation between different phyla nor is one nested in another, the TPI tree does not support, nor conflict with, the commonly accepted higher-order eukaryotic relationships [27]. Therefore, it seems likely that the TPI genes in all eukaryotes studied here including *Trimastix *were inherited vertically from the last common ancestor of eukaryotes.

#### Glyceraldehyde-3-phosphate dehydrogenase (GAPDH)

**Figure 2:**
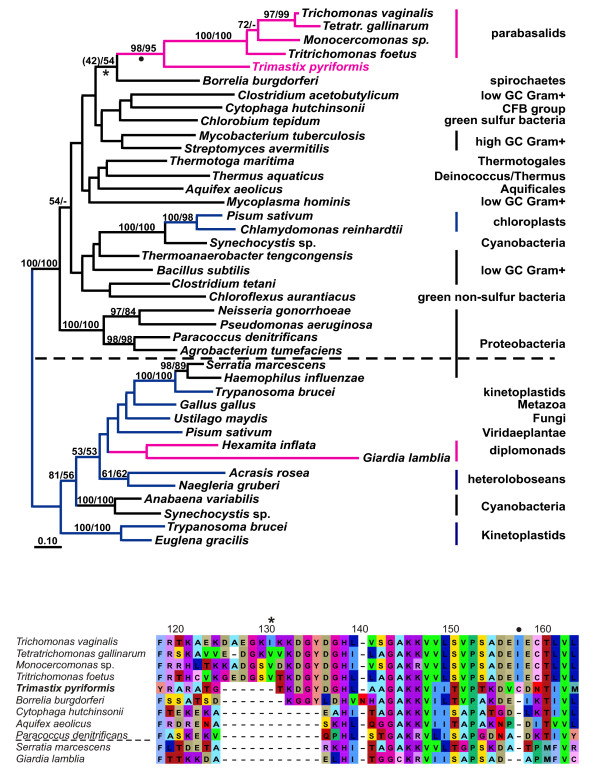
**A – ML tree of GAPDH protein sequences**. ML tree constructed from bacterial GapAB (above dashed line) and eukaryote cytosolic GapC (below dashed line) homologs (271 aligned aa positions). Note the bacterial type GapAB of plant chloroplasts as the result of a transfer from the cyanobacterial ancestor. The dot and the asterisk mark indel events supporting the branching of the parabasalid bacterial type GapAB with *Trimastix*. Colour coding and labelling as in (1A) plus eukaryotes being labelled in blue. Bootstrap values below 50% are shown where indicative of a potential LGT between bacteria and amitochondriate eukaryotes (*Borrelia *branching with *Trimastix *and parabasalids). The tree was rooted with cytosolic GapC homologs for display purposes. **B – Indels in the GAPDH protein sequence alignment**. Positions 119 to 164 of the GAPDH amino acid alignment are shown here to demonstrate insertions that support the branching of *Trimastix *and the parabasalids (1 aa – indicated by a dot) and of the spirochaete *Borrelia *with *Trimastix *and parabasalids (6–11 aa indicated by an asterisk). The dashed line separates GapAB (top) from GapC (bottom) sequences.

The phylogenetic relationships of GAPDHs have been studied in detail in a wide variety of organisms and the evolutionary history of this enzyme has turned out to be very complex [10,19,20,34]. For simplicity, the GAPDHs may be divided into two general groups, the eukaryote cytosolic GapC group and the bacterial GapAB group (Fig. 2A; [1,34]). Green plants contain in addition to their cytosolic GapC homolog, GapA and GapB duplicates which they acquired from a single ancestral gene in the ancestral chloroplast endosymbiont and which is used in the Calvin Cycle [1]. In diplonemids (a group of Euglenozoa) an obscure additional GapAB gene of a different origin was detected [34]. In parabasalids, no GapC has ever been found. They instead possess bacterial-like GapAB homologs which are located in the cytosol and are active in glycolysis [10,19]. Curiously, in our phylogenetic analyses, the GAPDH sequence of *Trimastix pyriformis *is the sister of the GAPDH of parabasalids (Fig. 2A) supported by high bootstrap values. The tight parabasalid/*Trimastix *clade is loosely associated with the sequence of the spirochaete *Borrelia burgdorferi *(bootstrap support 42%) similar to earlier reports of the affinities of the parabasalid homologs [20]. Despite the poor bootstrap support for this grouping, a common origin for the parabasalid, *Trimastix *and spirochaete homologs is confirmed by a unique homologous insertion, which is 11 amino acids long in parabasalids, but shorter in *Trimastix *and *Borrelia *(Fig. 2B). Another characteristic found in the *Trimastix *GAPDH is a eubacterial-like sequence in the S-loop of the enzyme, which is also seen in *Trichomonas *[19] and the other parabasalids (not shown).

#### Phosphoglycerate kinase (PGK)

**Figure 3:**
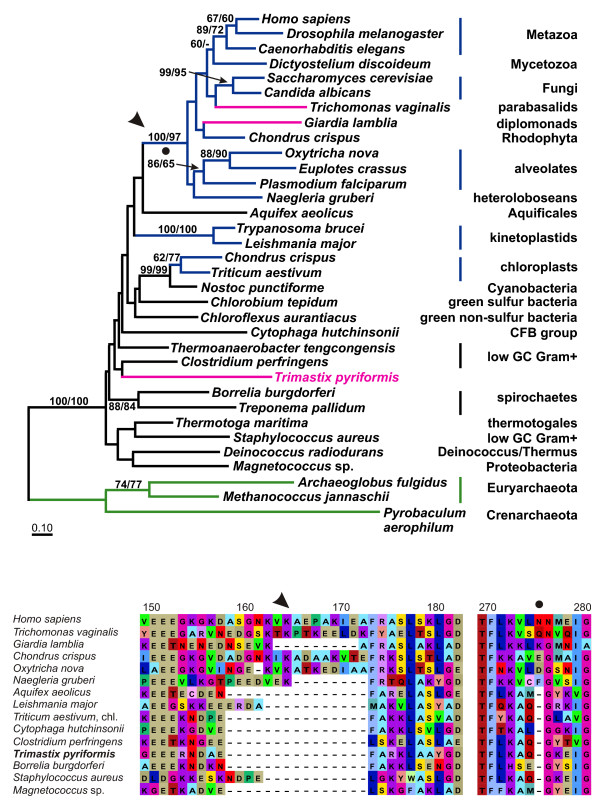
**A – ML tree of PGK protein sequences**. Maximum likelihood tree from PGK sequences of eukaryotes, eubacteria and archaebacteria (green labelling) based on 277 aligned aa positions. Color coding and labelling is as in Figures 1A and 2A. Possible chloroplast isoforms (*Chondrus *and *Triticus*) have not been directly localized, but their branching with cyanobacteria receives high bootstrap support. The presence of a surface loop (see arrowhead) and a 1 aa insertion (see black dot) unites all eukaryotes with the exception of kinetoplastids, *Trimastix *and the chloroplast homologs (see also Fig. 3B). For display purposes the tree has been rooted with archaebacteria. **B – Alignment of deduced PGK amino acid sequences**. Two sections of a PGK alignment are shown from eukaryotes and eubacteria. The first section (position 150–183) shows an insertion which produces a typical eukaryotic surface loop in the PGK protein (see also Fig. 3A) whereas the second section shows a 1 aa insertion at position 276. Both these features are not present in *Trimastix*, the kinetoplastids and the chloroplast homologs excluding these from the well supported remaining eukaryotes.

PGKs of archaebacteria, bacteria and eukaryotes are homologous [1]. With few exceptions, eukaryotic PGK genes constitute a monophyletic group nested within the bacterial clade (Fig. 3A). The structure of the bacterial subtree is not resolved and only few bacterial groups are recovered. In an earlier study, PGKs of kinetoplastids were located basal in the eukaryotic clade to the exclusion of chloroplast and bacterial PGKs [35]. However, with a broader sampling of bacterial PGKs, the homologs in kinetoplastids are separated from the major eukaryote clade by the sequence of *Aquifex *(Fig. 3A) and the chloroplast targeted PGKs branch with those from cyanobacteria. The PGK of *Trimastix pyriformis *also falls within the bacterial PGKs but its sister lineage can not be determined exactly. A relationship to *Clostridium perfringens *(Fig. 3A) or to *Deinococcus radiodurans *is suggested (not shown) but without any statistical support. A possible monophyly of the *Trimastix *and kinetoplastids PGKs can not be excluded entirely. In contrast, the PGKs of the amitochondriates *Giardia lamblia *and *Trichomonas vaginalis *are part of the eukaryotic clade. PGKs of the eukaryotic type are supposed to have a typical surface loop, which is shortened in bacterial PGKs [1]. In accordance with its phylogenetic position, the loop is missing completely in the PGK of *Trimastix *(Fig. 3B). It is present but shorter in the PGKs of kinetoplastids and the bacterium *Staphylococcus aureus*. On the other hand, the loop is also shorter in the PGKs of the protists *Naegleria gruberi *and *Giardia lambia *which branch in the eukaryotic clade. A one amino acid insertion is typical for PGKs of eukaryotes and not found in PGK of bacteria, kinetoplastids, chloroplasts and *Trimastix *(Fig. 3B; position 276 in the alignment). In summary, the PGK of *Trimastix *appears to be of different origin than PGKs of most eukaryotes and likely derives from an LGT event, although a bacterial donor lineage can not be determined.

#### Phosphoglycerate mutase (PGAM)

**Figure 4:**
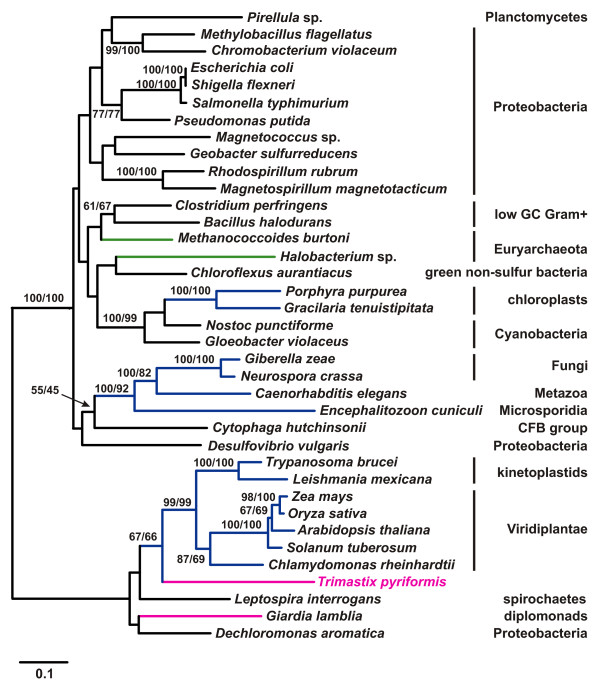
**ML tree of cofactor-independent PGAM protein sequences**. Phylogenetic tree of iPGAM sequences of eukaryotes, eubacteria and archaebacteria based on 394 aligned aa positions. Color coding and labelling as in Figures 1A and 2A, archaebacteria are labelled with green branches. Bootstrap values below 50% are shown where indicative of LGT events between eubacteria and eukaryotes. Note that the tree is split into two parts with high bootstrap support and that eukaryotes are in both subtrees. The tree was rooted arbitrarily.

Two types of PGAM can be distinguished that seem to be evolutionarily unrelated: a cofactor-dependent form (dPGAM) found in vertebrates and yeasts and a cofactor-independent form (iPGAM), mainly found in plants, algae and invertebrates [1,36]. Several eubacteria possess both forms and recently it has been shown that archaebacteria also have both forms [36-38]. *Trimastix *harbors a cofactor-independent PGAM, which groups together with the two kinetoplastid and the plant sequences (Fig. 4) in a cluster that also contains the amitochondriate *Giardia lamblia*, a spirochaete (*Leptospira*), and a proteobacterium (*Dechloromonas*). *Giardia *branches together with *Dechloromonas*, whereas *Leptospira *branches at the base of the *Trimastix*/plant/kinetoplastid clade. Although bootstrap support is very high for the whole cluster, it is not clear if both bacterial sequences are involved in LGT events or only one of them. The main cluster of the tree in Fig. 4 comprises prokaryotes and several eukaryotic sequences (Fungi, Metazoa, plant chloroplasts). Within that cluster, the two red algal chloroplast sequences branch with high bootstrap support with cyanobacteria as expected given the endosymbiotic origin of chloroplasts. The remaining eukaryotes from this part of the tree (Fungi, Metazoa, Microsporidia) branch in a cluster with a proteobacterium and *Cytophaga*, but bootstrap support is very low. Our tree suggests that iPGAM was involved in numerous lateral gene transfer events within all major groups of organisms including archaebacteria, eubacteria, and the eukaryotes. Eukaryotes seem to have acquired iPGAM several times independently, however weak or missing bootstrap support leaves it open which sequences were derived from which donor group.

#### Enolase

The enolases of eukaryotes are more closely related to archaebacteria than to bacteria (see Additional file 4), [39-41] and it was concluded that the enolase genes were transmitted vertically from the archaebacterial ancestors to eukaryotes [39,41]. The only exceptions are the cytosolic and probably plastid-targeted enolases of *Euglena gracilis*, which are missing a eukaryote-specific indel, and cluster among bacterial homologs with the spirochaete *Treponema pallidum *(not shown) [39]. Within the eukaryotic clade, intracellular transfer of enolase genes from the eukaryotic endosymbiont to the nucleus of the host cell was detected in chlorarchniophytes and cryptophytes [42]. On the subgenic level, insertions were probably transmitted between alveolates and land plants, two distantly related lineages, by lateral transfer and fine-scale recombination, resulting in a mosaic gene [42]. Parabasalids are the deepest branch in the eukaryotic tree (Additional file 4, [40]), but the discovery that a two amino acid deletion which was believed to be unique for Parabasalia is in fact a polymorphic character for this group does not support the hypothesis that they are the earliest diverging eukaryotes [40,43]. The enolase of *Trimastix pyriformis *is recovered as related to kinetoplastids with moderate bootstrap support, however the relationship of this clade to other eukaryotic taxa is not well resolved despite the fact that the eukaryote grouping itself is strongly supported (Additional file 4). Given this phylogeny, it is simplest to assume that *Trimastix *inherited its enolase gene by vertical descent.

#### Pyruvate kinase (PK)

**Figure 5:**
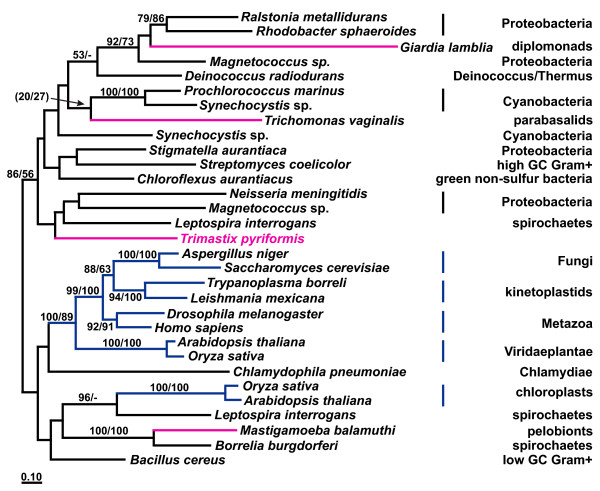
**ML tree of PK protein sequences**. Phylogenetic tree constructed from ATP dependent pyruvate kinases (PK) based on 297 aligned aa positions. Color coding and labelling is as in Figures 1A and 2A, except bootstrap values below 50% are shown where indicative of LGT events between eubacteria and eukaryotes (the branching of *Trichomonas *and cyanobacteria). Note that the four amitochondriate protists do not branch together. The root of the tree was chosen arbitrarily for display purposes.

Phylogenetic trees of PKs divide the bacterial sequences into two main clusters (Fig. 5, [44]). This split topology may be explained by an ancient gene duplication [44] or a complex LGT scenario. In any case, cytoplasmic PKs of fungi, kinetoplastids, animals, and plants form a tight clade, which is embedded in one of the bacterial clusters. By contrast, the PKs of the amitochondriate protists have a different origin: the PK of *Mastigamoeba balamuthi *is in the same subtree as the cytoplasmatic PKs, but it is closely related with an enzyme of *Borrelia burgdorferi*, a relationship that is supported by 100% bootstrap values. The PKs of *Trimastix pyriformis*, *Giardia lamblia*, and *Trichomonas vaginalis *fall within the second subtree, however, the branching order within this subtree is not well resolved. The enzyme from *Giardia*, although highly divergent in its amino acid sequence, very strongly groups with proteobacterial homologs to the exclusion of the other eukaryotes. Both the *Trichomonas *and the *Trimastix *sequences emerge in separate parts of this subtree, but the internal branching order depends on methods of analysis and is poorly supported in all cases. Thus although we cannot reconstruct the history of the *Trimastix *and *Trichomonas *genes with any precision, it does seem likely that they acquired their PK homologs in at least one event of LGT from a eubacterium, probably separately from *Giardia*.

#### Pyruvate-phosphate dikinase (PPDK)

In our phylogenetic analyses of diverse PPDKs sequences, the chloroplast targeted PPDKs from plants and a PPDK of a stramenopile form a tight clade to the exclusion of PPDKs of bacteria, archaebacteria, kinetoplastids, and amitochondriate protists (see Additional file 5, [45,46]). The PPKDs of protists are not monophyletic; the two possible PPDK genes of *Trichomonas vaginalis *are very similar and branch as sistergroup to the PPDKs of the Gram positive (high GC) bacteria. PPDKs of the amoeboid amitochondriates *Entamoeba histolytica *and *Mastigamoeba balamuthi *form a well-supported clade, which surprisingly branches as sistergroup with the PPDK of the proteobacterium *Methylococcus capsulatus *which in turn branches with the hyperthermophilic archaeon *Pyrobaculum aerophilum*, a grouping that was recently shown also by Slamovits and Keeling [23]. While the relationship of the kinetoplastid PPDKs within the tree is not fully resolved, they branch as the sister group to proteobacteria and *Chlorobium *plus *Bacteroides*, but receive no statistical support. *Trimastix pyriformis *and the two *Streblomastix *sequences form a well supported clade which in turn branches together with *Giardia lamblia*, with low to moderate support. Overall our tree does not support a common origin of all amitochondriate PPDKs as the parabasalid and the amoebozoan homologs are each strongly allied with a distinct prokaryotic group. However, the placement of the *Trimastix *PPDK with oxymonads and the diplomonad in an unresolved portion of the tree prevents definitive conclusions to be made regarding its origins but is suggestive of an LGT event to a common ancestral excavate.

## Discussion

### Phylogenetic patterns in glycolytic enzymes of amitochondriates

The extent of lateral gene transfer among glycolytic enzymes in *Trimastix pyriformis *is comparable with that in *Giardia lamblia*, *Trichomonas vaginalis*, and *Entamoeba histolytica*. At least four of the ten glycolytic enzymes that we identified in the ESTs of *Trimastix *(FBA, GAPDH, PGK, and PK), have been acquired from a bacterium by LGT and the phylogenies of two more enzymes (PGAM and PPDK) suggest LGTs as well. Although we propose that these events happened in the direction of prokaryote to eukaryote we cannot completely exclude the possibility that the opposite has happened, where a eukaryote donated a gene to a prokaryote. For instance, for some of these enzymes (e. g. FBA, Fig. 1A) the backbone phylogeny of prokaryotes is scrambled relative to accepted taxonomic relationships implying that gene transfers must have been occurring between these lineages as well. However, there are several reasons why we suspect most of the examples we discuss are more likely prokaryote-to-eukaryote transfers. The existing literature on LGT indicates that well substantiated cases of eukaryote to prokaryote transfer are rare, possibly because of difficulties in prokaryotes accommodating eukaryotic gene structure (e. g. introns/exons) [47]. Furthermore, in some of the cases that we discuss, the number of eukaryotes that possess homologs of an enzyme sub-type is very small relative to the number of prokaryotes and the eukaryotes are often distantly separated on the tree (e. g. FBA Fig. 1A). In other cases, such as GAPDH (Fig. 2A), the putative eukaryotic recipients of the transfers are distantly related to major eukaryotic clades nested deep within a group of prokaryotic and organellar homologs. For the glycolytic enzymes we have examined, the number of inter-domain LGT events required to explain the data are far fewer if eukaryotes are the recipients and prokaryotes the donor.

Although the trees supported separation of the eukaryote lineages and their emergence within predominantly prokaryotic clades, we could not determine the bacterial donor lineage implicated because of the poorly resolved branching patterns in the trees. Taking also the other amitochondriates into account, there are only few examples where we can trace back the donor lineages. Spirochaetes seem to be involved quite often in LGT events (Figs. 1A, 2A, 5) as was previously noted [20] and there was a conspicuous lack of putative LGT events between archaebacteria and eukaryotes. This is curious because LGTs between archaebacteria and amitochondriates are not exceptional and have been reported for enzymes catalyzing the reactions downstream from the glycolytic pathway involved in the fermentation of pyruvate [48-51].

Another curious pattern is that two enzymes in *Trimastix *that were clearly of vertical origin – TPI and enolase – were also apparently inherited vertically in the other amitochondriate eukaryotes (Additional Files 3 and 4). This indicates that the selection of enzymes in glycolysis that are replaced by LGTs in amitochondriate (and other) eukaryotes is not random. Selection for particular properties of these enzymes in amitochondriate protists must play some kind of role in this process, although precisely what these properties are remains a complete mystery.

### Alliances between 'excavate' homologs

In our trees, glycolytic enzyme homologs from excavates tend to cluster together to some extent (Figs 1A, 2A, 3A, Additional Files 2 and 5). However, the relationships between excavate homologs in the different enzyme trees are mutually contradictory.

Reconciling these groupings with known excavate relationships is a complex undertaking, especially given the uncertainty in the validity of an 'Excavata' clade [30]. Nevertheless, multiple gene phylogenies support several robust groupings of excavates, of which three are relevant here: (1) diplomonads plus parabasalids (and other taxa collectively known as Metamonada); (2) *Trimastix *and oxymonads (Preaxostyla); and (3) Euglenoids and kinetoplastids (Euglenozoa) [30]. If we assume these groupings are correct, then we can suggest plausible gene transfer scenarios.

For instance, the type II GPI of diplomonads and parabasalids is explained by a gene transfer event in their common 'metamonad' ancestor, while all other eukaryotes acquired their type I GPI homologs in one (or possibly two) separate event from a eubacterial donor (Additional file 2). On the other hand, the FBA tree suggests a common ancestor of diplomonads and *Trimastix *inherited an LGT of the class II type B enzyme to the exclusion of parabasalids (Fig. 1A). In this case, a common ancestor of the Preaxostyla and Metamonada may have acquired the FBA enzyme by LGT from eubacteria, with a second separate event replacing the enzyme later in parabasalids. In this scenario the kinetoplastids may retain the original 'excavate' class I FBA (like other eukaryotes), while a separate LGT must be postulated to explain why *Euglena *and yeasts possess the type A version of the class II enzyme [14].

The GAPDH tree indicates that *Trimastix *and parabasalids share a 'eubacterial' type enzyme whereas diplomonads and Euglenozoa have the canonical 'eukaryotic' versions of the enzyme (Fig. 2A). In this case it seems likely that either *Trimastix *or an ancestral parabasalid acquired the enzyme from a spirochaete-like donor and 'passed' this version to the other eukaryote by LGT. Evidence for such eukaryote-eukaryote LGT events is beginning to accumulate [51] and makes ecological sense if ancestors of these lineages coexisted in anoxic environments.

Finally, in the PPDK tree *Trimastix *groups with the oxymonad *Streblomastix *and the diplomonad *Giardia*, while the parabasalids branch independently among the high-GC gram positive bacteria (Additional file 5). As both of these clades emerge from within the bacteria it seems possible that either an ancestral 'excavate' lineage acquired the PPDK from a eubacterial donor with the parabasalids replacing their version later by an additional LGT event from eubacteria or *Trimastix *or the diplomonads acquired PPDK from the other by LGT. In either scenario, two LGT events are required.

The foregoing interpretations, while reasonable, are *ad hoc *explanations of observed branching patterns and insertion/deletion characters. Alternative LGT scenarios that account for the data may be equally plausible. However, to test these scenarios more information of two sorts is sorely needed. First, full genome sequences from the taxa represented in the tree are needed to determine if the homologs we have analyzed are the only members of the enzyme family present in the genome; if multiple copies exist in these genomes, then the phylogenetic histories of the copies may provide clues as to which ones are 'ancestral' and which are newly acquired by LGT. Second, a much better sampling of protistan and eubacterial genomes related to those implicated in the gene transfer events could provide key information regarding when such gene transfers took place, and allow the direction of transfer to be discerned. Indeed, a recent investigation aimed at broadening taxonomic sampling was able to provide much more precise information regarding the timing of LGT events in eukaryotic genomes [51].

### Can phylogenetic error be the explanation for the aberrant branching patterns?

As in every phylogenetic analysis, we may encounter random and systematic error. The lack of resolution in our trees can not be avoided due to the limited amount of data. Bootstrap values allow us at least to judge the statistical significance of bipartitions and determine which nodes are unresolved. We did not compare different tree topologies by likelihood ratio tests since this is too time consuming to complete for multiple topologies and the 10 enzyme families we examined. It was more important for us to determine if a gene from an amitochondriate branches within the main eukaryotic cluster than to trace back the actual bacterial donor lineage. For rather ancient LGTs, identifying the donor lineage might be impossible given the high background of bacterial LGTs, as pointed out by others [47,52].

We included some sequences in our analyses which did not pass the chi-squared tests for homogeneity of amino acid frequencies as implemented in TREE-PUZZLE (Table 2) and are therefore prone to phylogenetic artefacts. In most cases, these sequences branched with closely-related organisms in regions of the tree that were irrelevant to our conclusions. One exception is the PK of *Giardia lamblia *that is indeed represented by a very long branch and has significantly deviant amino acid composition (Table 2). However, it clusters with the short branches of proteobacterial PKs (Fig. 5) that are themselves not compositionally biased, suggesting that this grouping is likely historical rather than artefactual.

**Table 2:** Overview of the datasets used in phylogenetic reconstructions

dataset	number of sequences	number of aa	alpha^1^	sequences with divergent aa composition^2^
hexokinase	28	263	0.98	*Treponema denticola*
glucose phosphate isomerase	40	431	0.78	*Plasmodium falciparum* *Dictyostelium discoideum* *Aquifex aeolicus*
fructose bisphosphate aldolase	25	268	0.67	*Aquifex aeolicus*
triose phosphate isomerase	31	211	0.62	*Aquifex aeolicus* *Dictyostelium discoideum*
glyceraldehyde phosphate dehydrogenase	40	271	0.64	
phosphoglycerate kinase	34	277	0.67	
phosphoglycerate mutase	37	394	0.86	*Desulfovibrio vulgaris* *Gracilaria tenuistipitata* *Porphyra purpurea* *Encephalitozoon cuniculi* *Halobacterium *sp.
enolase	33	336	0.70	
pyruvate kinase	31	297	0.64	*Giardia lamblia*
pyruvate phosphate dikinase	29	682	0.51	*Rickettsia prowazekii* *Thermobifida fusca*

^1 ^the gamma shape parameter of the gamma distribution estimated using TREE-PUZZLE

^2 ^based on the chi square tests for deviation of amino acid frequencies implemented in TREE-PUZZLE

Not only is there conspicuous lack of evidence for 'phylogenetic artefacts' accounting for the aberrant branching patterns we describe, in a number of cases (Figs 1, 2, 3) we have also found characteristic insertion and deletion patterns in the enzyme homologs that support aspects of the phylogeny and the inference of LGT. As these regions were always removed from the data prior to the phylogenetic analyses and are not subject to phylogenetic 'artefacts' of the same sort as the trees, we suggest that the strongly supported phylogenetic patterns that we report likely represent the true history of the protein families.

### What about ancient paralogy and differential loss?

Provided that the phylogenies were correctly inferred, therefore, the unexpected relationships can be explained generally either by proposing LGT events or by ancient gene duplications followed by selective gene loss events in independent lineages [47]. However, the hallmarks of such 'paralogy scenarios' are multiple copies of enzymes in many taxa that generate 'mirror' organismal trees with random taxonomic gaps reflecting differential gene loss. Our phylogenies do not show this pattern. Thus if ancient paralogy were invoked to explain our data, it would require positing hypothetical ancestral organisms that retained progressively more and more paralogs of the enzymes in question as one moves deeper in the tree of life. Furthermore, cataclysmic numbers of differential losses of these paralogs would have to occur on independent lineages to generate the extant pattern. This scenario seems unlikely at best. However, although we do not see any evidence for paralogy, it is possible that some mixture of paralogy/gene loss and LGT events have generated the observed distribution of enzyme types in eukaryotes. Once again, full genome sequences from a much greater diversity of organisms are required to evaluate the relative likelihood of these alternative scenarios.

## Conclusion

### Replacement of glycolytic enzymes by LGT: selection or neutral evolution?

Do *Trimastix pyriformis *and other amitochondriates gain any advantages by replacing the canonical eukaryotic versions of the glycolytic enzymes or is it just a random process [53]? The use of pyrophosphate-linked instead of ATP-dependent enzymes can increase the overall efficiency of glycolysis, which is especially important if ATP production is solely carried out by glycolysis [54-56]. Pyrophosphate (PPi) is created during biosynthetic polymerization reactions and, in most organisms is hydrolyzed by inorganic pyrophosphatase in order to thermodynamically favor the anabolic reactions. Organisms like *Giardia lamblia*, which presumably lack inorganic pyrophosphatase, can use pyrophosphate instead of ATP as the phosphor-donor in some reactions [54,55]. In *Trimastix *we found in addition to an ATP-dependent PK, the pyrophosphate-linked PPDK as a second enzyme that catalyzes the conversion of phosphoenolpyruvate (PEP) to pyruvate (PPDK can also work in the other direction towards gluconeogenesis). For *Giardia*, in vitro tests suggested that both PK and PPDK play a role in glycolysis [57]. For *Trichomonas*, on the other hand, only PK activity could be detected [58] but the preliminary TIGR database of the *Trichomonas *genome contains two (very similar) PPDK genes (Fig. 5). Like in *Trimastix*, the mere presence of these two genes allows no conclusions to be made regarding their metabolic function. However, one could speculate that only one of the two is active in glycolysis, whereas the other one is still present in the genome but on its way to losing its function. It is noteworthy that we found over 30 times more ESTs for PPDK than for PK in the *Trimastix *library suggesting that the expression of PPDK is significantly higher in *Trimastix *than that of PK. The closely related oxymonad *Streblomastix strix *has also been shown to harbour PPDK (two copies in fact), but an ATP-dependent PK has not yet been found [23]. PPDK is also found in kinetoplastids, where it is involved in pyrophosphate recycling [59], and in plants, where it is involved in fixation of CO_2 _[60]. Our tree suggests that the PPDKs of all amitochondriates sampled here have been acquired by lateral gene transfer events. It is tempting to assume that the acquisition of this PPi-linked enzyme is the result of an adaptation to the amitochondriate nature of these organisms, thus enabling them to increase ATP yield during glycolysis.

Interestingly, laterally acquired glycolytic enzymes of amitochondriates were shown to have biochemical properties more similar to the bacterial homologs than to the canonical eukaryotic enzymes consistent with their phylogenetic position [19,58]. In *Trichomonas *PK is stimulated by ribose 5-phosphate and glycerate 3-phosphate, as is the case for many bacteria, while most eukaryotic PKs are allosterically activated by fructose 1,6-bisphosphate [57,58]. Similarly, the *Trichomonas *GAPDH exhibits sensitivity to the inhibitor koningic acid at comparable levels to eubacterial homologs and two orders of magnitude less than eukaryotic GAPDHs. This sensitivity is directly linked to the sequence of the S-loop domain of the enzyme which has a eubacterial signature in *Trichomonas *[19] and *Trimastix*. A selective advantage may be hidden behind these differences but it is not yet recognized. The same is true for FBAs of amitochondriates, which are all of class II and have been acquired in several independent LGT events. Thus, it seems that laterally transferred genes often retain some of their 'bacterial properties', although the biochemical properties of these enzymes need to be studied in much more detail.

The most abundant pattern of LGT in eukaryotes is the transfer of genes from the bacterial ancestors of the modern mitochondrion and plastid to the cell nucleus during the process of organelle genome reduction [11,47,53]. Several proteins involved in the energy metabolism of eukaryotes are sister to or nested within alpha-proteobacteria in phylogenetic inferences which is seen as straightforward evidence for mitochondrion-to-host gene transfer [61]. Some go further and regard every affiliation of a eukaryotic protein to any bacterial phylum as probable endosymbiotic gene transfer [52,62]: first, given the extent of LGT and gene loss among bacteria, the respective homolog may no longer be found in extant alpha proteobacteria; or second, during the course of the gene transfer from endosymbiont to the host genome, the gene had no functional constraints and may have acquired numerous mutations which now mask its origin. Plants recruited a huge number of their nuclear genes from the plastid genome as revealed by a comparison of the genomes of *Arabidopsis thaliana*, cyanobacteria, and chloroplasts [63]. Massive endosymbiotic gene transfers from eukaryote to eukaryote were observed from the nucleomorph (the reduced nucleus of the eukaryotic endosymbiont) to the nucleus of the mixotrophic alga *Bigelowiella natans *[64]. The extent of LGT from non-organellar (in contrast to endosymbiotic organelle) donors to eukaryotic genomes seems to vary from lineage to lineage. For rumen ciliates, diplomonads, *Bigelowiella natans*, and *Trimastix pyriformis *(this study), it was shown that they acquired a significant number of genes by LGT from different bacterial, and even eukaryotic, lineages [64-66]. More sporadic, but steadily increasing in number, are documented LGTs to other protist lineages [11,13,48,50,51,65,66]. The pattern that seems to emerge is that LGT is not infrequent in phagotrophic protists, while its impact on the evolution of other eukaryotic lineages is either minor or simply not yet known [64,66]. A mechanism has been proposed, where small pieces of DNA of the engulfed bacteria escape digestion and are incorporated into the protist genome replacing the ancestral eukaryotic genes over time in a rather random manner [53]. As already discussed, the high frequency of LGT in amitochondriates may be partially but not entirely caused by the fact that these protists live phagotrophically. Selection as a result of adaptation to certain environments might favor the uptake of specific proteins more suitable to the host organism, making LGT a non-random process [66]. Yet, the relative importance of random neutral evolution versus the effect of selection in causing LGT in eukaryotes is something we can not answer until we understand more about the general frequency of LGT in eukaryotic microbes and the functional properties of the enzymes involved.

## Methods

### Source of sequences and alignments

All sequences obtained in our EST project from *Trimastix pyriformis *were used for searches against the non-redundant NCBI database (BLAST, [67]) and cDNA clones with high similarity to 10 glycolytic enzymes were found. Sequencing of all clones was completed using the primer walking method. Truncated 5' ends of cDNAs were amplified using the GeneRacer Kit (Invitrogen). Length and G+C contents of the coding regions plus accession numbers of the deduced protein sequences are summarized in table 1.

Sequences homologous to the glycolytic enzymes of *Trimastix *were retrieved from the NCBI non-redundant protein database. The PGK, TPI, PK, and PPDK sequences for *Trichomonas vaginalis *were downloaded from the TIGR database [68]. The TPI dataset was complemented with the TPI sequences of *Entamoeba histolytica*, *Spironucleus barkhanus*, and *Acrasis rosea *(accession numbers EF064144–EF064146), which were obtained by PCR using degenerate primers directed against the N-terminal motifs VGGNWK (TF-1) and VGGNFK (TF-2) and the C-terminal VGGASL (TR-1). PCRs were carried out with genomic DNA using standard methods. The PGK sequence of *Naegleria gruberi *was obtained from another EST project (accession number EF064143).

The individual datasets were aligned with ClustalW using default settings [69] and subsequently adjusted manually. An alignment of enolases, to which we manually added the *Trimastix *sequence, was kindly provided by Patrick Keeling (University of British Columbia, Vancouver). Regions of ambiguous alignment were excluded from further analyses. Care was taken that each dataset contained a reasonable number of sequences representing all major taxonomic groups and sequence clades. The number of sequences and amino acid positions used for phylogenetic analyses of each dataset are listed in table 2.

### Phylogenetic analyses

Maximum likelihood (ML) phylogenies were inferred using PMBML [70], with the JTT substitution model and a mixed four category discrete gamma-model of among-site variation. The gamma shape parameter alpha was estimated using TREE-PUZZLE [71] (see table 2 for alpha values). TREE-PUZZLE also provided the distance matrix (using the same models) which was used to construct ML distance trees using the Fitch-Margoliash algorithm as implemented in FITCH from the Phylip package [72]. In both types of analyses, ten random additions with global rearrangements were used to find the optimal tree. ML distance bootstrap values for bipartitions were calculated by analyses of 100 resampled data sets using PUZZLEBOOT ([73] distributed by A. J. Roger and M. E. Holder). The alpha parameter for each resampled dataset was calculated separately using the same models as described above. Only one random sequence addition was done. ML bootstrap values were calculated with the program phyml [74], using a discrete gamma model with four rate categories and 100 resamplings; the gamma parameter alpha was calculated individually for each resampled dataset. All trees were caluclated unrooted but are shown with outgroups for display purposes.

## Abbreviations

EST, expressed sequence tags; LGT, lateral gene transfer; FBA, fructose-bisphosphate aldolase; GPI, glucose phosphate isomerase; GAPDH, glyceraldehyde-3-phosphate dehydrogenase; PFK, phosphofructokinase; PPDK, pyruvate phosphate dikinase; PK, pyruvate kinase; TPI, triosephosphate isomerase; PGK, phosphoglycerate kinase; PGAM, phosphoglycerate mutase; Ppi, pyrophosphate; PEP, phosphoenolpyruvate; ML, maximum likelihood.

## Authors' contributions

AS and MB did most of the molecular biology work, phylogenetic analyses and drafted the manuscript. JDS cultured *Trimastix *and established the cDNA library and participated in the sequence alignments. AJR conceived of and supervised this study and edited the manuscript. All authors read and approved the final manuscript.

## Supplementary Material

Additional File 1 **ML tree of hexokinase protein sequences**. Phylogenetic tree of hexokinase sequences derived with maximum likelihood (alignment of 263 aa positions). Amitochondriate protists are labelled pink, the residual eukaryotes are labelled blue. Eubacteria are labelled black. The numbers on the bipartitions are ML distance bootstrap values (puzzleboot) on the left and ML bootstrap values (phyml) on the right. Bootstrap values below 50% are omitted. The grouping of *Trimastix *with alveolates and kinetoplastids receives no support. The tree was rooted with the eubacterial homologs for display purposes. Click here for file

Additional File 2 **ML tree of type I and II GPI protein sequences**. Maximum likelihood tree based on 431 aligned aa positions from type I and type II GPIs from eukaryotes and eubacteria. The top (main) part of the tree are type I GPIs and below the dashed line are type II GPIs. Color coding and labelling as in Additional file 1. Note that *Trimastix *has a type I GPI unlike the other amitochondriates (parabasalids and diplomonads). Type II GPIs were used to root the tree for display purposes. Click here for file

Additional File 3 **ML tree of TPI protein sequences**. ML tree of eubacterial and eukaryotic TPI sequences (211 aligned aa positions). Eukaryotes are monophyletic for TPI with high bootstrap support, however the relationships of different eukaryote groups are not resolved. The amitochondriate protists do not branch together but are dispersed throughout the eukaryotes. Color coding and labelling as in Additional file 1. The tree is rooted with eubacterial homologs for display purposes. Click here for file

Additional File 4 **ML tree of enolase protein sequences**. ML tree constructed from archaebacterial, eubacterial and eukaryote enolase sequences based on 336 aligned aa positions. Parabasalids branch independently from the other amitochondriate taxa at the base of the eukaryote part. Eukaryotes are monophyletic with high bootstrap support. Color coding and labelling as in Additional file 1. Archaebacteria are labelled green. The tree was rooted with eubacterial and archaebacterial homologs for display purposes. Click here for file

Additional File 5 **ML tree of PPDK protein sequences**. ML tree constructed from an alignment of 674 aa positions from archaebacterial, eubacterial and eukaryote PPDK sequences. Note the strong support for the grouping of the parabasalids with low GC Gram positives and of the Amoebozoa with a proteobacterium and an archaebacterium. Color coding and labelling as in Additional file 1. Archaebacteria are labelled green. The root of the tree was chosen at the split of the chloroplast and stramenopile sequences from the remaining homologs for display purposes. Click here for file

## References

[B1] Fothergill-Gilmore LA, Michels PA (1993). Evolution of glycolysis. Prog Biophys Mol Biol.

[B2] Muller M (1988). Energy metabolism of protozoa without mitochondria. Annu Rev Microbiol.

[B3] Muller M (1993). The hydrogenosome. J Gen Microbiol.

[B4] Muller M, Coombs, GH., Vickerman, K., Sleigh, MA. and Warren A (1998). Enzymes and compartmentation of core energy metabolism of anaerobic protists: a special case in eukaryotic evolution.. Evolutionary relationships among protozoa.

[B5] Tovar J, Fischer A, Clark CG (1999). The mitosome, a novel organelle related to mitochondria in the amitochondrial parasite Entamoeba histolytica. Mol Microbiol.

[B6] Williams BA, Hirt RP, Lucocq JM, Embley TM (2002). A mitochondrial remnant in the microsporidian Trachipleistophora hominis. Nature.

[B7] Tovar J, Leon-Avila G, Sanchez LB, Sutak R, Tachezy J, van der Giezen M, Hernandez M, Muller M, Lucocq JM (2003). Mitochondrial remnant organelles of Giardia function in iron-sulphur protein maturation. Nature.

[B8] Muller M, G. H. Coombs KVMASAW (1998). Enzymes and compartmentation of core energy metabolism of anaerobic protists - a special case inm eukaryotic evolution?. Evolutionary relationships among protozoa.

[B9] Mertens E, Ladror US, Lee JA, Miretsky A, Morris A, Rozario C, Kemp RG, Muller M (1998). The pyrophosphate-dependent phosphofructokinase of the protist, Trichomonas vaginalis, and the evolutionary relationships of protist phosphofructokinases. J Mol Evol.

[B10] Viscogliosi E, Muller M (1998). Phylogenetic relationships of the glycolytic enzyme, glyceraldehyde-3-phosphate dehydrogenase, from parabasalid flagellates. J Mol Evol.

[B11] Henze K, Horner DS, Suguri S, Moore DV, Sanchez LB, Muller M, Embley TM (2001). Unique phylogenetic relationships of glucokinase and glucosephosphate isomerase of the amitochondriate eukaryotes Giardia intestinalis, Spironucleus barkhanus and Trichomonas vaginalis. Gene.

[B12] Muller M, Lee JA, Gordon P, Gaasterland T, Sensen CW (2001). Presence of prokaryotic and eukaryotic species in all subgroups of the PP(i)-dependent group II phosphofructokinase protein family. J Bacteriol.

[B13] Wu G, Henze K, Muller M (2001). Evolutionary relationships of the glucokinase from the amitochondriate protist, Trichomonas vaginalis. Gene.

[B14] Sanchez L, Horner D, Moore D, Henze K, Embley T, Muller M (2002). Fructose-1,6-bisphosphate aldolases in amitochondriate protists constitute a single protein subfamily with eubacterial relationships. Gene.

[B15] Bapteste E, Moreira D, Philippe H (2003). Rampant horizontal gene transfer and phospho-donor change in the evolution of the phosphofructokinase. Gene.

[B16] Marsh JJ, Lebherz HG (1992). Fructose-bisphosphate aldolases: an evolutionary history. Trends Biochem Sci.

[B17] Henze K, Morrison HG, Sogin ML, MullerM (1998). Sequence and phylogenetic position of a class II aldolase gene in the amitochondriate protist, Giardia lamblia. Gene.

[B18] Patron NJ, Rogers MB, Keeling PJ (2004). Gene replacement of fructose-1,6-bisphosphate aldolase supports the hypothesis of a single photosynthetic ancestor of chromalveolates. Eukaryot Cell.

[B19] Markos A, Miretsky A, Muller M (1993). A glyceraldehyde-3-phosphate dehydrogenase with eubacterial features in the amitochondriate eukaryote, Trichomonas vaginalis. J Mol Evol.

[B20] Figge RM, Cerff R (2001). GAPDH gene diversity in spirochetes: a paradigm for genetic promiscuity. Mol Biol Evol.

[B21] Hrdy I, Mertens E, Nohynkova E (1993). Giardia intestinalis: detection and characterization of a pyruvate phosphate dikinase. Exp Parasitol.

[B22] Reeves RE (1968). A new enzyme with the glycolytic function of pyruvate kinase. J Biol Chem.

[B23] Slamovits CH, Keeling PJ (2006). Pyruvate-phosphate dikinase of oxymonads and parabasalia and the evolution of pyrophosphate-dependent glycolysis in anaerobic eukaryotes. Eukaryot Cell.

[B24] O'Kelly CJ, Farmer MA, Nerad TA (1999). Ultrastructure of Trimastix pyriformis (Klebs) Bernard et al.: similarities of Trimastix species with retortamonad and jakobid flagellates. Protist.

[B25] Bernard C (2000). Some free-living flagellates (Protista) from anoxic habitats.. Ophelia.

[B26] Brugerolle G (1997). Ultrastructure of Trimastix convexa Hollande, an amitochondriate anaerobic flagellate with a previously undescribed organization.. European Journal of Protistology.

[B27] Simpson AG, Roger AJ (2004). The real 'kingdoms' of eukaryotes. Curr Biol.

[B28] Dacks JB, Silberman JD, Simpson AG, Moriya S, Kudo T, Ohkuma M, Redfield RJ (2001). Oxymonads are closely related to the excavate taxon Trimastix. Mol Biol Evol.

[B29] Simpson AG (2003). Cytoskeletal organization, phylogenetic affinities and systematics in the contentious taxon Excavata (Eukaryota). Int J Syst Evol Microbiol.

[B30] Simpson AG, Inagaki Y, Roger AJ (2006). Comprehensive multigene phylogenies of excavate protists reveal the evolutionary positions of "primitive" eukaryotes. Mol Biol Evol.

[B31] Hampl V, Horner DS, Dyal P, Kulda J, Flegr J, Foster PG, Embley TM (2005). Inference of the phylogenetic position of oxymonads based on nine genes: support for metamonada and excavata. Mol Biol Evol.

[B32] Cardenas ML, Cornish-Bowden A, Ureta T (1998). Evolution and regulatory role of the hexokinases. Biochim Biophys Acta.

[B33] Nowitzki U, Flechner A, Kellermann J, Hasegawa M, Schnarrenberger C, Martin W (1998). Eubacterial origin of nuclear genes for chloroplast and cytosolic glucose-6-phosphate isomerase from spinach: sampling eubacterial gene diversity in eukaryotic chromosomes through symbiosis. Gene.

[B34] Qian Q, Keeling PJ (2001). Diplonemid glyceraldehyde-3-phosphate dehydrogenase (GAPDH) and prokaryote-to-eukaryote lateral gene transfer. Protist.

[B35] Adje CA, Opperdoes FR, Michels PA (1998). Molecular analysis of phosphoglycerate kinase in Trypanoplasma borreli and the evolution of this enzyme in kinetoplastida. Gene.

[B36] Galperin MY, Bairoch A, Koonin EV (1998). A superfamily of metalloenzymes unifies phosphopentomutase and cofactor-independent phosphoglycerate mutase with alkaline phosphatases and sulfatases. Protein Sci.

[B37] Fraser HI, Kvaratskhelia M, White MF (1999). The two analogous phosphoglycerate mutases of Escherichia coli. FEBS Lett.

[B38] van der Oost J, Huynen MA, Verhees CH (2002). Molecular characterization of phosphoglycerate mutase in archaea. FEMS Microbiol Lett.

[B39] Hannaert V, Brinkmann H, Nowitzki U, Lee JA, Albert MA, Sensen CW, Gaasterland T, Muller M, Michels P, Martin W (2000). Enolase from Trypanosoma brucei, from the amitochondriate protist Mastigamoeba balamuthi, and from the chloroplast and cytosol of Euglena gracilis: pieces in the evolutionary puzzle of the eukaryotic glycolytic pathway. Mol Biol Evol.

[B40] Keeling PJ, Palmer JD (2000). Parabasalian flagellates are ancient eukaryotes. Nature.

[B41] Tracy MR, Hedges SB (2000). Evolutionary history of the enolase gene family. Gene.

[B42] Keeling PJ, Palmer JD (2001). Lateral transfer at the gene and subgenic levels in the evolution of eukaryotic enolase. Proc Natl Acad Sci U S A.

[B43] Keeling PJ (2004). Polymorphic insertions and deletions in parabasalian enolase genes. J Mol Evol.

[B44] Schramm A, Siebers B, Tjaden B, Brinkmann H, Hensel R (2000). Pyruvate kinase of the hyperthermophilic crenarchaeote Thermoproteus tenax: physiological role and phylogenetic aspects. J Bacteriol.

[B45] Nevalainen L, Hrdy I, Muller M (1996). Sequence of a Giardia lamblia gene coding for the glycolytic enzyme, pyruvate,phosphate dikinase. Mol Biochem Parasitol.

[B46] Maldonado RA, Fairlamb AH (2001). Cloning of a pyruvate phosphate dikinase from Trypanosoma cruzi. Mol Biochem Parasitol.

[B47] Andersson JO (2005). Lateral gene transfer in eukaryotes. Cell Mol Life Sci.

[B48] Field J, Rosenthal B, Samuelson J (2000). Early lateral transfer of genes encoding malic enzyme, acetyl-CoA synthetase and alcohol dehydrogenases from anaerobic prokaryotes to Entamoeba histolytica. Mol Microbiol.

[B49] Sanchez LB, Galperin MY, Muller M (2000). Acetyl-CoA synthetase from the amitochondriate eukaryote Giardia lamblia belongs to the newly recognized superfamily of acyl-CoA synthetases (Nucleoside diphosphate-forming). J Biol Chem.

[B50] Suguri S, Henze K, Sanchez LB, Moore DV, Muller M (2001). Archaebacterial relationships of the phosphoenolpyruvate carboxykinase gene reveal mosaicism of Giardia intestinalis core metabolism. J Eukaryot Microbiol.

[B51] Andersson JO, Hirt RP, Foster PG, Roger AJ (2006). Evolution of four gene families with patchy phylogenetic distributions: influx of genes into protist genomes. BMC Evol Biol.

[B52] Schnarrenberger C, Martin W (2002). Evolution of the enzymes of the citric acid cycle and the glyoxylate cycle of higher plants. A case study of endosymbiotic gene transfer. Eur J Biochem.

[B53] Doolittle WF (1998). You are what you eat: a gene transfer ratchet could account for bacterial genes in eukaryotic nuclear genomes. Trends Genet.

[B54] Reeves RE (1976). How useful is the energy in inorganic pyrophosphate?. Trends Biochem Sci.

[B55] Mertens E (1993). ATP versus pyrophosphate: glycolysis revisited in parasitic protists. Parasitol Today.

[B56] Mertens E (1991). Pyrophosphate-dependent phosphofructokinase, an anaerobic glycolytic enzyme?. FEBS Lett.

[B57] Park JH, Schofield PJ, Edwards MR (1997). Pyruvate kinase is present in Giardia intestinalis. Exp Parasitol.

[B58] Mertens E, Van Schaftingen E, Muller M (1992). Pyruvate kinase from Trichomonas vaginalis, an allosteric enzyme stimulated by ribose 5-phosphate and glycerate 3-phosphate. Mol Biochem Parasitol.

[B59] Acosta H, Dubourdieu M, Quinones W, Caceres A, Bringaud F, Concepcion JL (2004). Pyruvate phosphate dikinase and pyrophosphate metabolism in the glycosome of Trypanosoma cruzi epimastigotes. Comp Biochem Physiol B Biochem Mol Biol.

[B60] Matsuoka M (1995). The gene for pyruvate, orthophosphate dikinase in C4 plants: structure, regulation and evolution. Plant Cell Physiol.

[B61] Canback B, Andersson SG, Kurland CG (2002). The global phylogeny of glycolytic enzymes. Proc Natl Acad Sci U S A.

[B62] Esser C, Ahmadinejad N, Wiegand C, Rotte C, Sebastiani F, Gelius-Dietrich G, Henze K, Kretschmann E, Richly E, Leister D, Bryant D, Steel MA, Lockhart PJ, Penny D, Martin W (2004). A genome phylogeny for mitochondria among alpha-proteobacteria and a predominantly eubacterial ancestry of yeast nuclear genes. Mol Biol Evol.

[B63] Martin W, Rujan T, Richly E, Hansen A, Cornelsen S, Lins T, Leister D, Stoebe B, Hasegawa M, Penny D (2002). Evolutionary analysis of Arabidopsis, cyanobacterial, and chloroplast genomes reveals plastid phylogeny and thousands of cyanobacterial genes in the nucleus. Proc Natl Acad Sci U S A.

[B64] Archibald JM, Rogers MB, Toop M, Ishida K, Keeling PJ (2003). Lateral gene transfer and the evolution of plastid-targeted proteins in the secondary plastid-containing alga Bigelowiella natans. Proc Natl Acad Sci U S A.

[B65] Andersson JO, Roger AJ (2002). A cyanobacterial gene in nonphotosynthetic protists--an early chloroplast acquisition in eukaryotes?. Curr Biol.

[B66] Ricard G, McEwan NR, Dutilh BE, Jouany JP, Macheboeuf D, Mitsumori M, McIntosh FM, Michalowski T, Nagamine T, Nelson N, Newbold CJ, Nsabimana E, Takenaka A, Thomas NA, Ushida K, Hackstein JH, Huynen MA (2006). Horizontal gene transfer from Bacteria to rumen Ciliates indicates adaptation to their anaerobic, carbohydrates-rich environment. BMC Genomics.

[B67] Altschul SF, Madden TL, Schaffer AA, Zhang J, Zhang Z, Miller W, Lipman DJ (1997). Gapped BLAST and PSI-BLAST: a new generation of protein database search programs. Nucleic Acids Res.

[B68] TIGR http://www.tigr.org/tdb/e2k1/tvg/.

[B69] Thompson JD, Gibson TJ, Plewniak F, Jeanmougin F, Higgins DG (1997). The CLUSTAL_X windows interface: flexible strategies for multiple sequence alignment aided by quality analysis tools. Nucleic Acids Res.

[B70] Veerassamy S, Smith A, Tillier ER (2003). A transition probability model for amino acid substitutions from blocks. J Comput Biol.

[B71] Schmidt HA, Strimmer K, Vingron M, von Haeseler A (2002). TREE-PUZZLE: maximum likelihood phylogenetic analysis using quartets and parallel computing. Bioinformatics.

[B72] Felsenstein J (2004). PHYLIP (Phylogeny inference package), version 3.6..

[B73] PUZZLEBOOT http://rogerlab.biochemistryandmolecularbiology.dal.ca/Software/Software.htm.

[B74] Guindon S, Gascuel O (2003). A simple, fast, and accurate algorithm to estimate large phylogenies by maximum likelihood. Syst Biol.

