# Supplementary figures and images for: The glycolytic pathway of Trimastix pyriformis is an evolutionary mosaic
Source: BMC Evol Biol. 2006 Nov 23;6:101. doi: 10.1186/1471-2148-6-101 (PMC1665464; doi:10.1186/1471-2148-6-101)

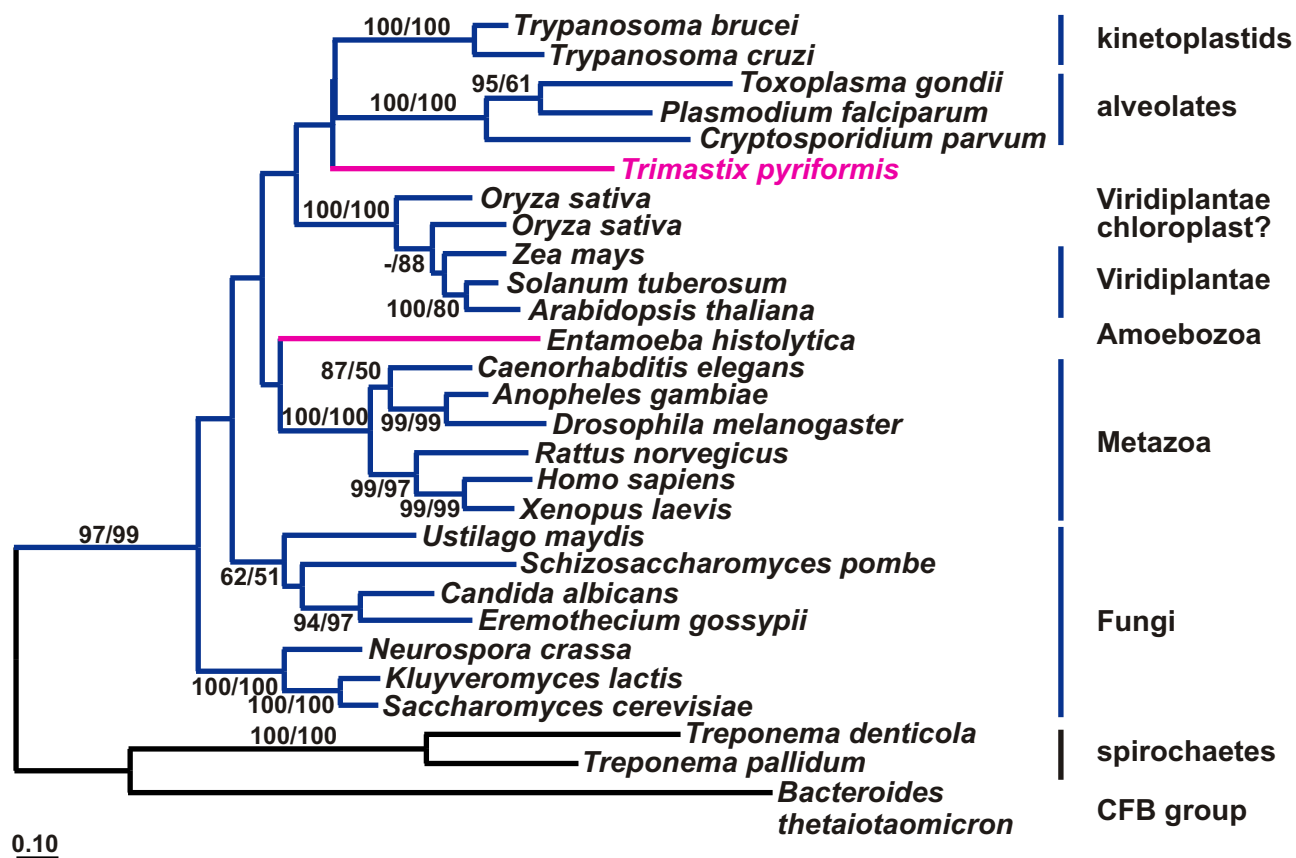

Additional File1- hexokinase

Supplement: Additional File 1 — ML tree of hexokinase protein sequences. Phylogenetic tree of hexokinase sequences derived with maximum likelihood (alignment of 263 aa positions). Amitochondriate protists are labelled pink, the residual eukaryotes are labelled blue. Eubacteria are labelled black. The numbers on the bipartitions are ML distance bootstrap values (puzzleboot) on the left and ML bootstrap values (phyml) on the right. Bootstrap values below 50% are omitted. The grouping of Trimastix with alveolates and kinetoplastids receives no support. The tree was rooted with the eubacterial homologs for display purposes. [file 1471-2148-6-101-S1.pdf]

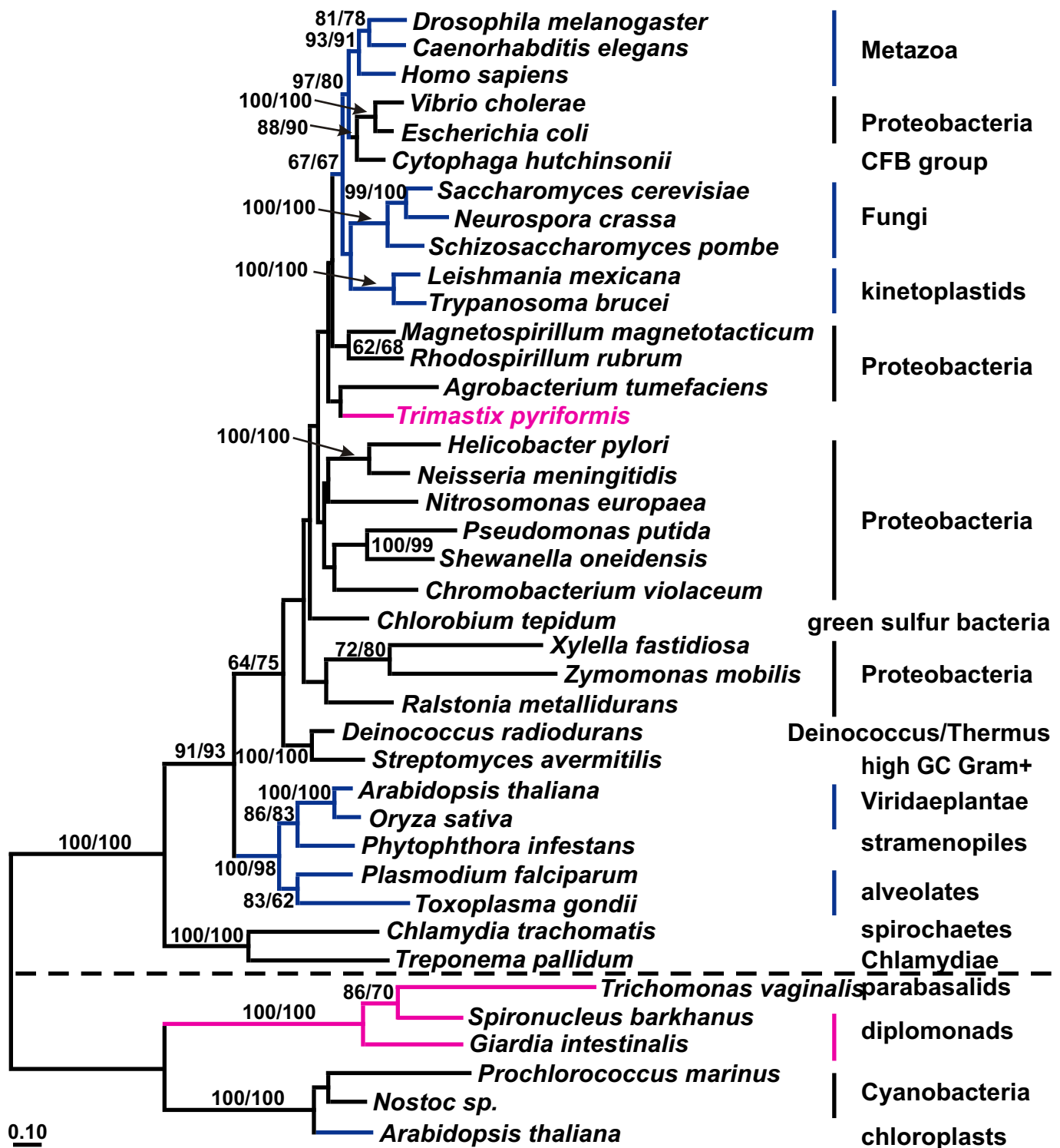

Additional File 2 - glucose-6-phosphate isomerase

Supplement: Additional File 2 — ML tree of type I and II GPI protein sequences. Maximum likelihood tree based on 431 aligned aa positions from type I and type II GPIs from eukaryotes and eubacteria. The top (main) part of the tree are type I GPIs and below the dashed line are type II GPIs. Color coding and labelling as in Additional file 1. Note that Trimastix has a type I GPI unlike the other amitochondriates (parabasalids and diplomonads). Type II GPIs were used to root the tree for display purposes. [file 1471-2148-6-101-S2.pdf]

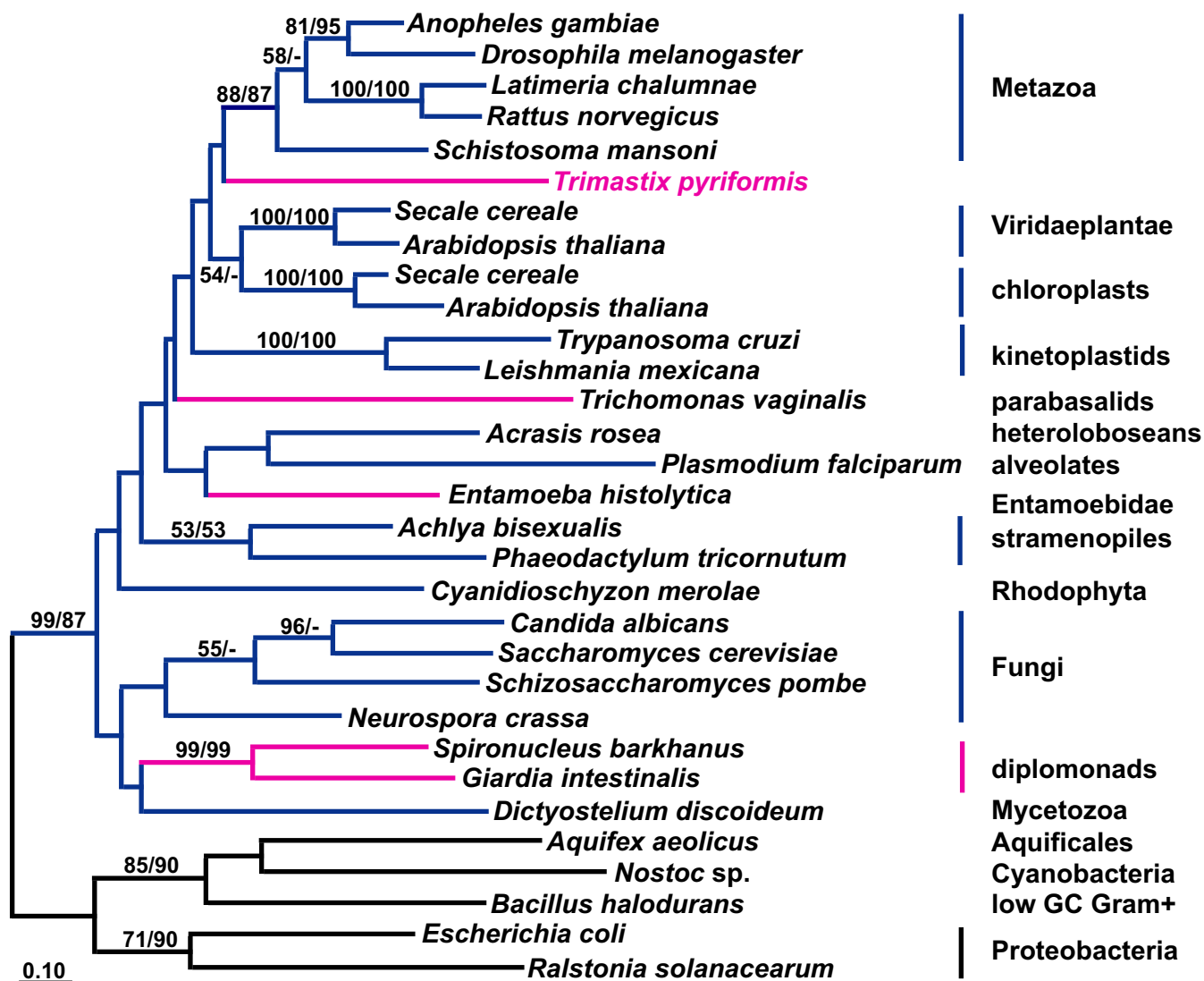

Additional File 3 - triosephosphate isomerase

Supplement: Additional File 3 — ML tree of TPI protein sequences. ML tree of eubacterial and eukaryotic TPI sequences (211 aligned aa positions). Eukaryotes are monophyletic for TPI with high bootstrap support, however the relationships of different eukaryote groups are not resolved. The amitochondriate protists do not branch together but are dispersed throughout the eukaryotes. Color coding and labelling as in Additional file 1. The tree is rooted with eubacterial homologs for display purposes. [file 1471-2148-6-101-S3.pdf]

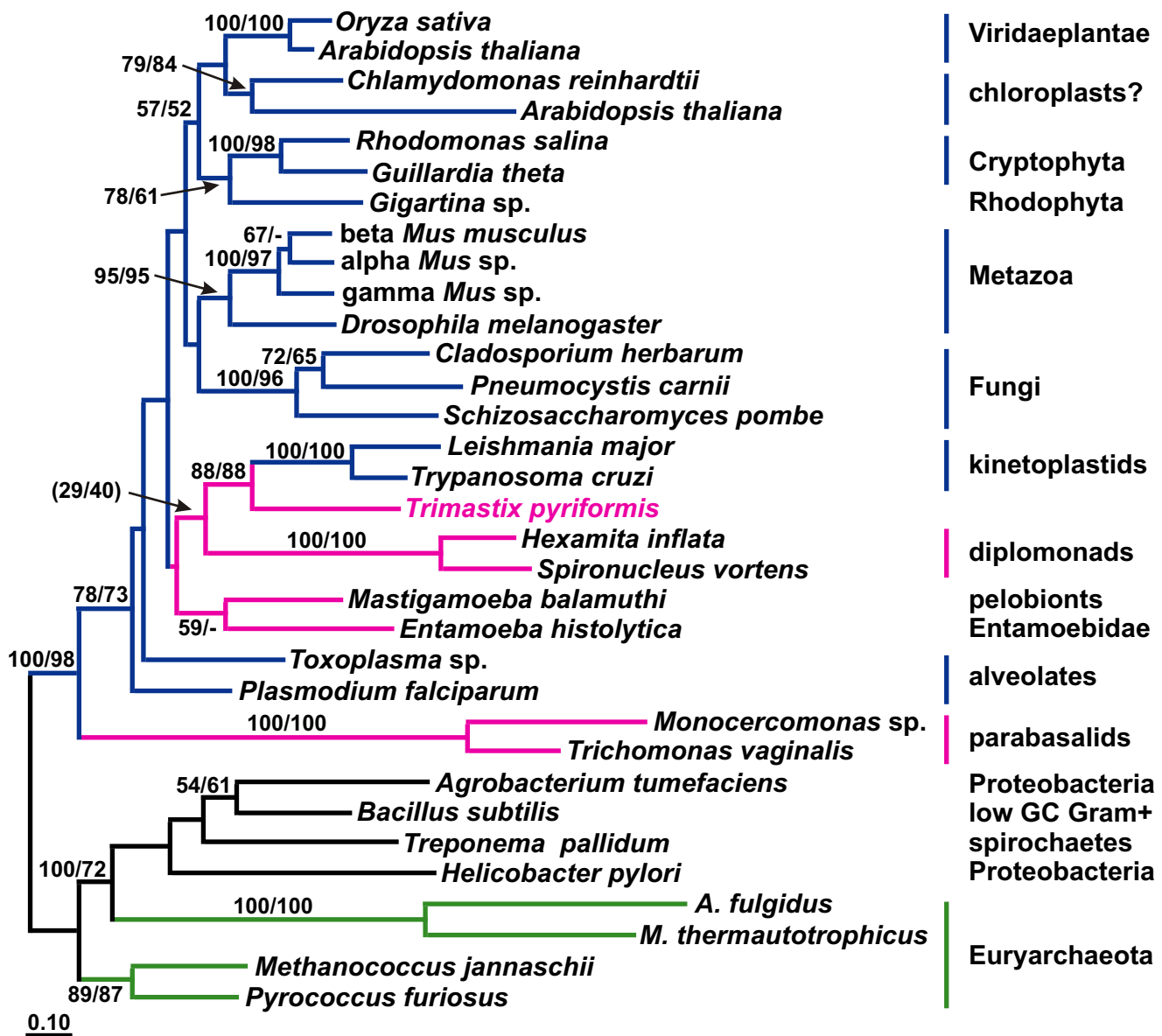

Additional File 4 - enolase

Supplement: Additional File 4 — ML tree of enolase protein sequences. ML tree constructed from archaebacterial, eubacterial and eukaryote enolase sequences based on 336 aligned aa positions. Parabasalids branch independently from the other amitochondriate taxa at the base of the eukaryote part. Eukaryotes are monophyletic with high bootstrap support. Color coding and labelling as in Additional file 1. Archaebacteria are labelled green. The tree was rooted with eubacterial and archaebacterial homologs for display purposes. [file 1471-2148-6-101-S4.pdf]

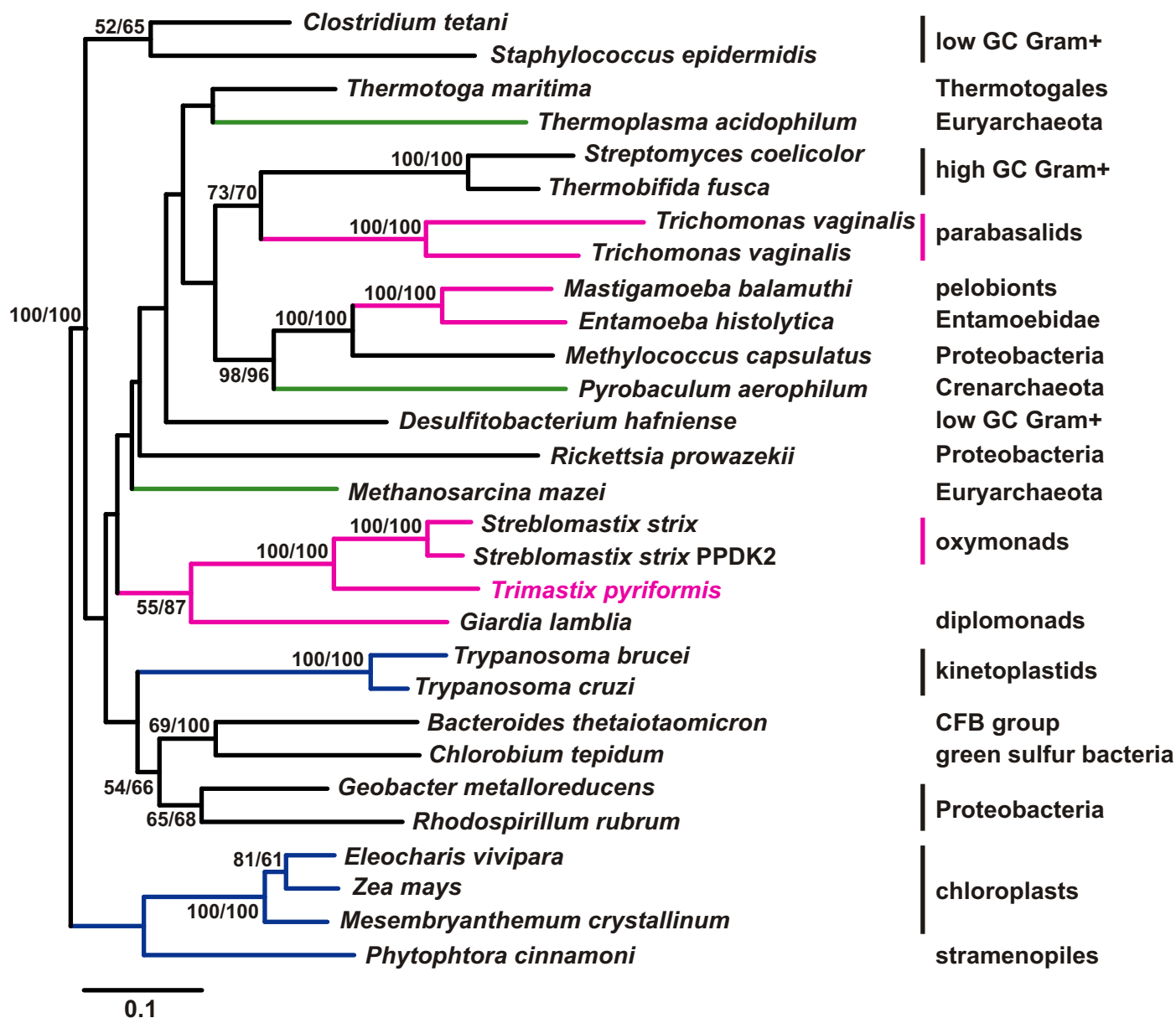

Additional File 5 - pyruvate phosphate dikinase

Supplement: Additional File 5 — ML tree of PPDK protein sequences. ML tree constructed from an alignment of 674 aa positions from archaebacterial, eubacterial and eukaryote PPDK sequences. Note the strong support for the grouping of the parabasalids with low GC Gram positives and of the Amoebozoa with a proteobacterium and an archaebacterium. Color coding and labelling as in Additional file 1. Archaebacteria are labelled green. The root of the tree was chosen at the split of the chloroplast and stramenopile sequences from the remaining homologs for display purposes. [file 1471-2148-6-101-S5.pdf]
